# A Bibliometric Review of Brain–Computer Interfaces in Motor Imagery and Steady-State Visually Evoked Potentials for Applications in Rehabilitation and Robotics

**DOI:** 10.3390/s25010154

**Published:** 2024-12-30

**Authors:** Nayibe Chio, Eduardo Quiles-Cucarella

**Affiliations:** 1 Instituto de Automática e Informática Industrial, Universitat Politècnica de València, 46022 Valencia, Spain; nachch@posgrado.upv.es; 2 Facultad de Ingeniería, Ingeniería Mecatrónica, Universidad Autónoma de Bucaramanga, Bucaramanga 680003, Colombia

**Keywords:** brain–computer interface, steady-state visually evoked potential, motor imagery, rehabilitation, robot arm, robot hand

## Abstract

In this paper, a bibliometric review is conducted on brain–computer interfaces (BCI) in non-invasive paradigms like motor imagery (MI) and steady-state visually evoked potentials (SSVEP) for applications in rehabilitation and robotics. An exploratory and descriptive approach is used in the analysis. Computational tools such as the biblioshiny application for R-Bibliometrix and VOSViewer are employed to generate data on years, sources, authors, affiliation, country, documents, co-author, co-citation, and co-occurrence. This article allows for the identification of different bibliometric indicators such as the research process, evolution, visibility, volume, influence, impact, and production in the field of brain–computer interfaces for MI and SSVEP paradigms in rehabilitation and robotics applications from 2000 to August 2024.

## 1. Introduction

Brain–computer interfaces (BCI) have been identified as one of the challenges to be solved in the field of robotics research [1]. There exist bibliometric reviews on BCI in the field of music [2], as well as analyses on the increase in the literature on or trends in BCI [3,4,5,6,7], rehabilitation robots with virtual reality and exoskeletons [8,9], strokes [5], BCI software [3], machine learning [10], motor imagery [10], external stimuli, events, and emotions [11].

Different databases have been used, such as Web of Science [5,6,7,8,10] and Scopus [10]. Bibliometric software analysis tools have been used, including CiteSpace [8], VOSViewer [2,5,9,11], Dervent Data Analyzer [6], R [7], and Scimat [2].

BCI is a research topic that emerged in the 1970s [12]. It is a system that translates brain signals into commands, enabling individuals to interact with their environment and communicate through external devices or applications [3,4,5,6,7]. Brain signals can be obtained actively (consciously), reactively (indirectly), or passively (unconsciously) [2]. These signals are used in motor imagery (MI) or steady-state visually evoked potential (SSVEP) paradigms. The MI paradigm involves imagining the performance of motor tasks without physically performing them [3,5,10], while the SSVEP paradigm uses visual or auditory stimuli at a specific frequency to elicit brain responses at the same frequency [4,6,7].

There are applications to improve the quality of a patient’s life in the context of stroke treatment and rehabilitation [4,5,6], device control [3,10], user status monitoring [3], games [3,4,10,11], cognitive enhancement [3,6], neuroscience [13], motor function recovery [10,13], marketing [10], education [10,11], military devices [11], and assistive medical devices [4,11].

The term ‘bibliometric’ is defined as “the application of mathematical and statistical methods to books and the communication media” [14]. Bibliometrics has two approaches. The first is a descriptive approach, entailing the description of the impact and productivity of a body of literature and the dissemination of its findings. The second approach is normative, meaning that it establishes norms, rules, and heuristics to demonstrate the intellectual progress of a given topic [15]. This study relies on the descriptive approach combined with a quantitative methodology. The bibliometric approach is different from a systematic literature review (SLR); it is an instrument used to collect information through a procedure that ensures the understanding, transparency, and replicability of the results [16,17], guaranteeing the quality and reliability of the research findings.

Table 1 identifies the most relevant studies and compares their metrics and parameters for the bibliometric analysis.

The documents in Table 1 do not contain any studies that meet the remit of this study. Therefore, this study performs a bibliometric review to identify documents on and authors conducting research on brain-computer interfaces in the fields of rehabilitation and robotics under the non-invasive paradigms of MI and SSVEP. The bibliometric analysis is conducted using the paradigms of MI and SSVEP because they are non-invasive methods used to interact with external devices, such paradigms sharing technologies and technological tools to acquire and process signals. The bibliometric analysis with respect to both paradigms allows to identify the evolution and trends in BCI applied to motor function rehabilitation and interaction with robotics devices. The MI paradigm is a non-invasive approach that activates areas of the brain used when performing real movements, stimulating neuroplasticity and helping control prosthetics, exoskeletons, and assistance robotic devices. The SSVEP paradigm is a non-invasive approach which, through visual stimuli, helps people with locked syndrome or paralysis communicate, allowing their interaction with external devices or virtual environments. The results identify the different concepts and the evolution of BCIs in the context of rehabilitation and robotics applications with respect to both paradigms.

## 2. Materials and Methods

A bibliometric review was conducted using bibliometric methods and tools in three phases. The first phase consisted of the bibliometric analysis to identify contributions within the areas of knowledge. Phase 2 consisted of an analysis conducted using the co-citation and co-occurrence maps generated with the VOSViewer tools version 1.6.20 and the biblioshiny application for R-Bibliometrix to RStudio version 4.4.1. A formulation of development areas is proposed in the second phase. Phase 3 consisted of analyzing information from the studies and identifying concepts and units of analysis.

Different tools were used, such as Scopus, the biblioshiny application for R-Bibliometrix, and VOSViewer. Before starting the first phase, the Elsevier database of bibliographic references and peer-reviewed literature citations, called Scopus [18], was consulted. The search in Scopus generated 566 documents, limited to a study’s title, abstract, and keywords. The search equation included the terms “brain computer interface”, “motor imager*”, “steady state visual* evoked potential”, “ssvep”, “rehabilitation robot*“, “robot* hand”, “robot* arm”, and “collaborative robot”. Only studies in English and Spanish were included, reducing the number of documents to 547 by excluding 19 documents.

For the first phase, the biblioshiny application of R-bibliometrix was used, and the metadata of the 547 documents were analyzed with respect to the abstract, document type, journal, language, publication year, title, total citations, author, affiliation, cited references, DOI, and keywords.

**Figure 1:**
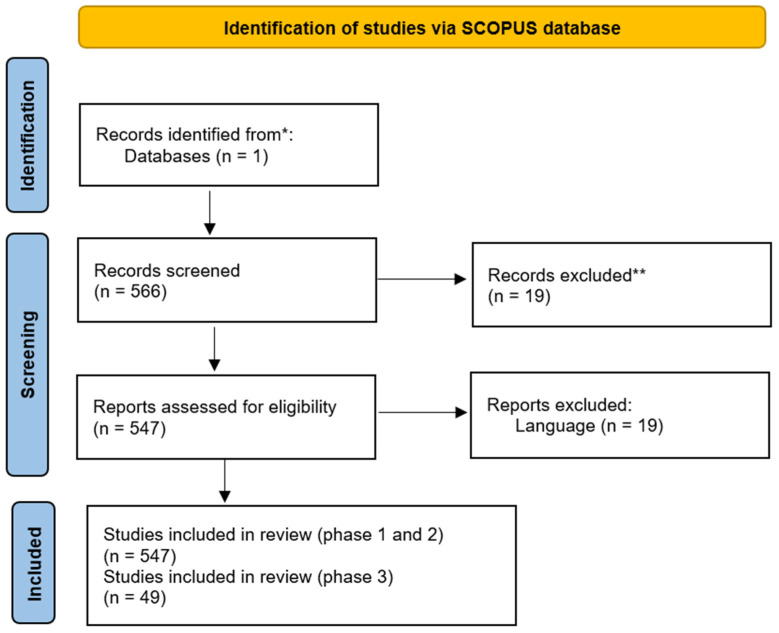
Information flow diagram on the bibliometric analysis. * Database: Scopus. ** Excluded by automation tool.

Figure 1 shows an adaptation of the PRISMA 2020 [19] flow diagram, which includes the search of the Scopus database.

A first analysis was conducted using information from Scopus and the biblioshiny application for R-bibliometrix related to the evolution of publications by year, sources with more studies, authors, affiliation, country, cited studies, and documents by topic.

In the second phase, an analysis of information relating to the authors, countries, references, and publication sources through the co-citation maps was performed; an analysis of the word co-occurrence maps was conducted and the thematic evolution was analyzed.

The biblioshiny application for R-bibliometrix was used to analyze thematic evolutions, authors’ co-citations, and the most influential studies investigating rehabilitation and robotics using the paradigms of motor imagination or steady-state visually evoked potentials in the context of brain–computer interface systems. The computational tool VOSViewer is a freely accessible software that generates bibliometric graphics to be used in support of citation, co-citation, and co-occurrence analyses [20]. This tool was used to perform the co-occurrence analysis of words.

In the third phase, 49 documents were selected from the 547 documents based on a specific indicator. The citation feature is a relevant indicator of quality, visibility, and scientific impact [21]. From the number of citations of a study, a factor is estimated for each article based on two indicators: the impact factor of the journal where it is published, taken from the SJR (Scimago Journal Rank), and the number of citations of the article. The estimator is then calculated using the expression I = C * (SJR + 1) (23). Through the Pareto analysis, the studies comprising 80% of the impact factor were included for a total of 49 documents to be evaluated from 2000 to 2024.

These phases, which rely on the use of bibliometrics, contribute to future systematic literature reviews and the development of state-of-the-art studies. Bibliometric analyses allow for the evaluation and analysis of academic production based on mathematical and statistical methods to classify and provide results from a set of documents.

## 3. Results

Below are the results generated by using the tools Scopus, the biblioshiny application for R-Bibliometrix, and VOSViewer. Table 2 shows the metadata analysis conducted with the biblioshiny application for R-Bibliometrix. The first filter was made with the R-Bibliometrix application to analyze the documents in terms of the following metadata: abstract, document type, journal, year of publication, and total citations. Each analyzed document yielded an excellent status with respect to the evaluation, indicating that all documents had this information. However, with respect to author, affiliation, cited reference, DOI, keyword plus, and keyword metadata, the status of the documents emerged as good or acceptable. Nevertheless, documents with such evaluation statuses can still be evaluated within this range. Therefore, it was decided to include all documents in the bibliometric analysis.

### 3.1. Evolution of Study Publications by Year

**Figure 2:**
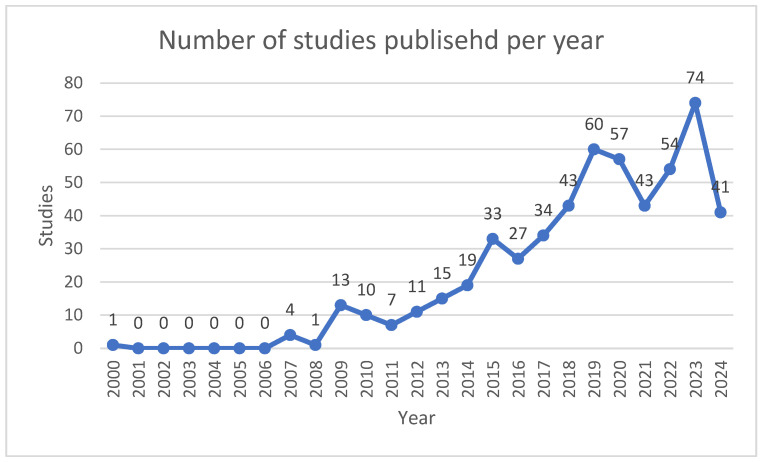
Number of studies published per year.

The evolution of published studies determines the relationship between the year and the number of studies. Figure 2 shows the number of studies published per year between 2000 and August 2024. Of the 547 studies, we found various types, including 261 scientific articles, 243 conference proceedings or proceedings, 12 book chapters, 29 reviews or review conferences, and two documents identified as data documents and editorials. This study identified 1718 authors, with eight publishing individually and 1710 collaborating. The study–author ratio was 0.318, with an average of 3.14 authors per study and a ratio of 4.98 co-authors. The international co-authorship percentage was 24.68%. The average number of citations per study was 26.01, with an annual growth rate of 16.73%.

Figure 2 shows the trend in the publication of studies, with the first study on the subject of BCI to use the paradigms of MI and SSVEP in the context of rehabilitation and robotics applications being published in 2000. No evidence of publication of such studies emerged from 2001 to 2006. Between 2007 and 2014, the range was 1–19, while, in 2015, the number of publications increased by 33; from 2018, more than 40 publications have been published, with the highest number of publications, i.e., 74, reached in 2023. It can be observed that there is a trend toward an increase in publications over time, indicating that there is a greater number of researchers who are studying this topic.

### 3.2. Publication Sources with More Studies

The main publication sources with five or more published studies are presented. Metrics are included to objectively and quantifiably evaluate the impact factor, quartile, and H index of the SJR.

Table 3 shows the publication sources of the studies, primarily in the engineering field. Three indicators are presented. The first is the quartile, which indicates the position of a publication source with respect to all publications in the same research area. The highest number of relevant publications was 26 in the Journal of Neural Engineering, followed by the IEEE Transactions on Neural Systems and Rehabilitation Engineering with 22 studies. Both were located in the Q1 quartile, which is occupied by the top 25% journals in the field. The third largest publication source was a conference proceeding with 21 studies, and the fourth was a book series in artificial intelligence and bioinformatics with 17 studies. The next indicator is the H index, which evaluates scientific journals (publication sources) based on the quantity and quality of the citations received. The sources with the highest index were Neuroimage, with an SJR-derived H index of 418, and Plos One, with an SJR-derived H index of 435, followed by Sensors with an SJR-derived H index of 245. The Journal of Neural Engineering, IEEE Transactions on Neural Systems and Rehabilitation Engineering, Frontiers in Human Neuroscience, Biomedical Signal Processing and Control, ACM International Conference Proceeding Series, and Frontiers in Neuroscience exhibited an SJR-derived H index between 100 and 200, indicating that they are the most outstanding sources of publications within this research field. Finally, the bibliometric indicator SJR (Scimago Journal Rank) is related to how the research area, quality, and reputation of the scientific journal have a direct impact on the value of the citation, with the 2023 SJR score of 2.44 for the Neuroimage source indicating that the citations of the articles in this journal have a high value.

### 3.3. Authors

**Figure 3:**
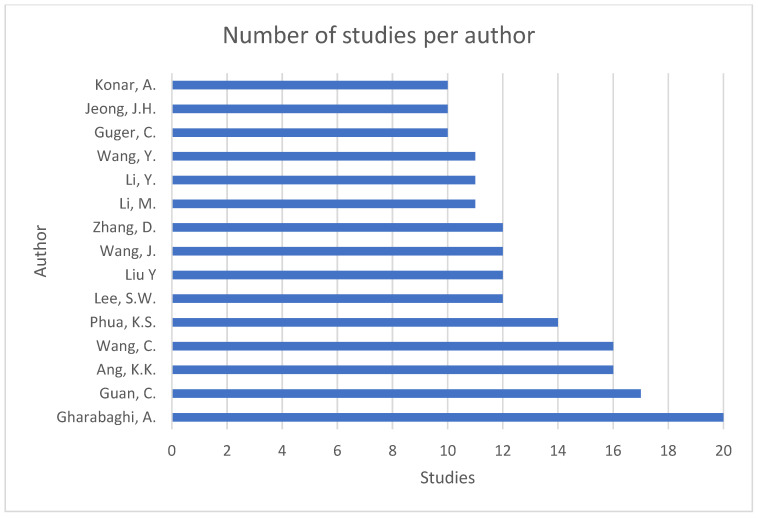
Number of studies per author.

Figure 3 shows the number of studies per author, presenting the authors with 10 or more studies.

**Figure 4:**
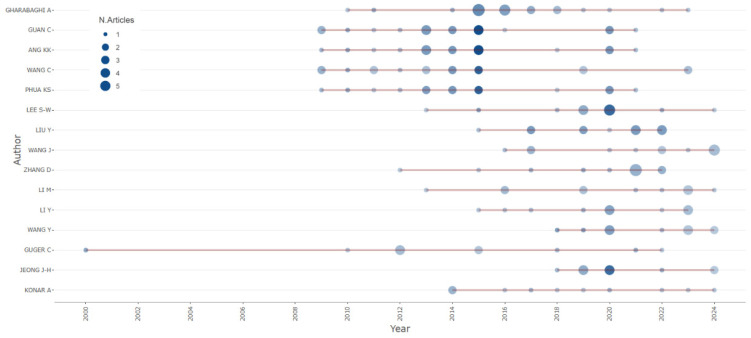
Author’s production over time.

Figure 4 shows the number of studies per author and year obtained from the biblioshiny application for R-bibliometrix, with the authors having 10 or more studies presented.

Figure 3 and Figure 4 show the most representative authors publishing on BCI under the MI or SSVEP paradigms in the context of rehabilitation and robotics applications. At the time of our study, the most influential author was Garabaghi, A. with 20 studies, followed by Guan, C., Ang, K.K., and Wang, C., each having published 16 or 17 studies between 2013 and 2016. Since 2020, other authors have contributed to this area, such as Wang, J., Li, M., Wang, Y., and Jeong, J-H, each having published 10 to 12 studies.

Table 4 shows the classification of the authors based on the H, G, and M indexes and the number of published studies. The H index is an author-level metric that seeks to measure the academic and productive impact of a scientific document cited by other authors [22]. Authors with an H index greater than 10 included Gharabaghi, A., Guan, C., Ang, K.K., Phua, K.S., and Wang, C. The G index is an indicator that quantifies scientific productivity [23], with the highest G index (i.e., 20) found for Gharabaghi, A. Finally, the M index is the ratio of the H index to the number of years elapsed since the first publication by the author in question [24], with the highest M index held by Gharabaghi, A., with 1.067 since 2010.

### 3.4. Affiliation

Table 5 shows the institutions associated with over 25 publications. Eberhard Karls University Tübingen, Germany, was found to lead with 51 published studies, followed by the Institute for Infocomm Research, Singapore, and Korea University, each with 41 studies. Two European institutions, Aristóteles University of Thessaloniki, Greece, and Miguel Hernández University of Elche, Spain, have contributed 26 studies each. Latin America, through the Federal University of Espirito Santo, Brazil, has contributed 38 studies, while four Chinese institutions have contributed 25 to 30 publications each: Shangai Jiao Tong University, South China University of Technology, Tianjin University, and Tiangin Technological University.

### 3.5. Country

**Figure 5:**
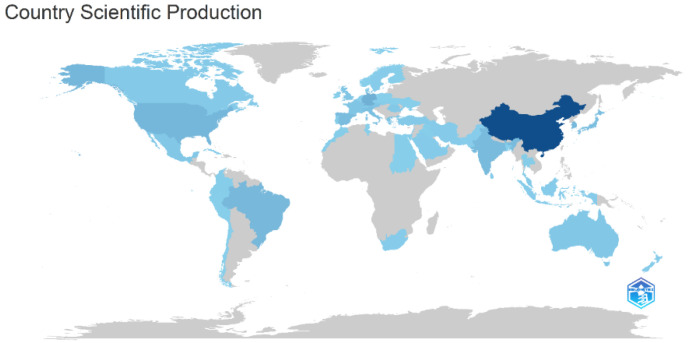
Visual representation of scientific production by country.

Figure 5 shows the distribution map of production by country, with the highest production in China, followed by the USA, Germany, India, Brazil, Japan, Spain, and South Korea. The map shown in Figure 5 is a visual representation of the amount of production by country. Shown in gray are the countries that, at the time of our study, did not have any published studies on the research topic under investigation. The blue color varies from dark to light, with the dark blue color indicating countries with the highest production levels. As the color degrades, the production intensity (i.e., quantity of published studies) decreases; the production intensity can be seen in Table 6.

Table 6 shows the scientific output by country, the authors’ nationality, and the countries with the most citations. At the time of our study, China had the highest number of scientific outputs and publishing authors, while Germany had the highest number of citations. The highest number of authors per country was found in China, with 127, followed by Germany, with 34 authors, and India, with 24 authors. In the range of 10–19 authors were countries such as the United States of America, Japan, Spain, South Korea, Brazil, Singapore, and Austria, while Italy had less than 10 authors. It can be concluded that the highest number of authors is in Asia and Europe, followed by Latin America and the United States of America. In terms of scientific production, China was found to be associated with 155 publications, followed by the United States of America with 47, Germany with 46, and India with 40. This indicates that scientific production is diversified across Europe, Asia, and North America.

### 3.6. Studies

Citation analysis, a method that counts the number of times an article has been cited in other works, is a tool for finding relevant information about a study in a particular thematic field [25]. Table 7 shows the most cited titles, revealing that the most cited title, at the time of our study, was “Quadcopter control in three-dimensional space using a noninvasive motor imagery-based brain-computer interface” by Lafleur K., Cassady K., Doud A., Shades K., Rogin E., and He B., published in 2013 in the Journal of Neural Engineering. The next three most cited articles are related to stroke, paraplegic, or tetraplegic patients.

### 3.7. Studies by Field of Knowledge

**Figure 6:**
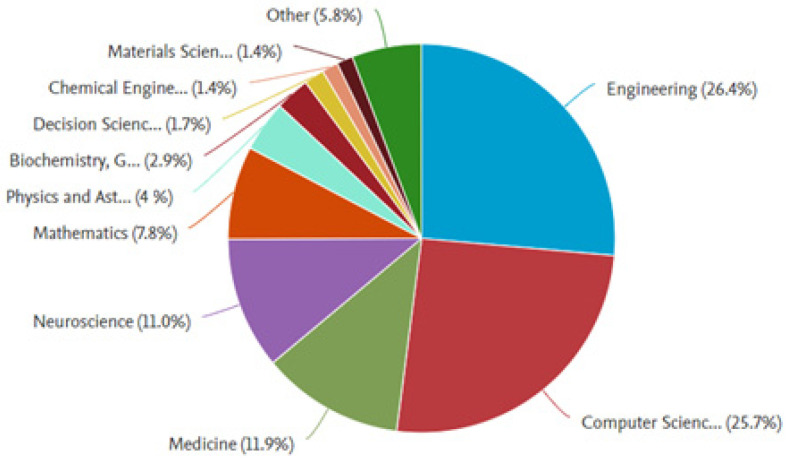
Percentage of studies by area of knowledge.

Figure 6 shows the percentage of studies that have been classified according to the area of knowledge, which includes engineering, computer science, medicine, neuroscience, mathematics, physics, biochemistry, decision sciences, chemistry, materials, and other fields of knowledge. The highest number of studies was found to have been published in the field of engineering (26.4%) and computer science (25.7%), amounting to 52.1% of the total studies. A total of 11% of the studies were classified as being representative of neuroscience and 11.9% of medicine, for a total of 22.9% of the studies classified in these areas of knowledge. A total of 25% of the available studies were found to be distributed among other areas, with percentages lower than 10%.

### 3.8. Co-Citation Network

Co-citation analysis measures the frequency with which two publications are cited together and allows us to determine the relationship between ideas and authors [26]. Proximity maps identify the strength of the relationship between two elements by assessing their distance, with a smaller distance indicating a stronger relationship, helping recognize groups of related elements [27].

#### 3.8.1. Author Co-Citation Network

**Figure 7:**
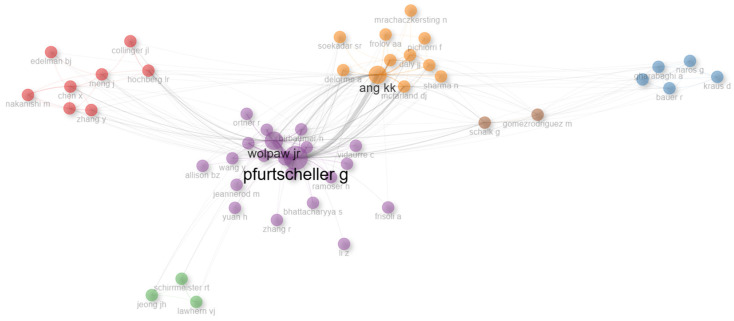
Visual representation of the co-citation network of a group of authors.

The first co-citation analysis was performed using the biblioshiny application for R-bibliometrix. Figure 7 shows a visual representation of the co-citation network of a group of authors, the nodes indicate the number of times they have been cited together with others. Authors Wolpaw, J.R., Pfurtscheller, G., and Ang, K.K. were found to be the authors of studies on motor imagery paradigms and visually evoked potentials in rehabilitation and robotics applications with the greatest influence. Each cluster is identified by a different color and indicates a research topic; if the distance between the nodes is small, it means that the studies are closely related, while distant nodes indicate that the authors have not been co-cited and that the studies deal with different research areas. Several color groups emerged with respect to co-cited authors, with three groups being particularly evident: dark green for Schalk, G., light green for Ang, K.K., blue for Wolpaw, J.R., and red for Pfurtscheller, G. There were two groups containing authors of great relevance and importance: yellow for Ang, K.K. and purple for Wolpaw, J.R. and Pfurtscheller, G. The other clusters had nodes of equal size, the blue cluster indicating Gharabaghi, A., the brown cluster indicating Schalk, G., the green cluster indicating Jeong, J.H., and the red cluster indicating Chen, X. The existence of groups of co-cited authors was determined.

**Figure 8:**
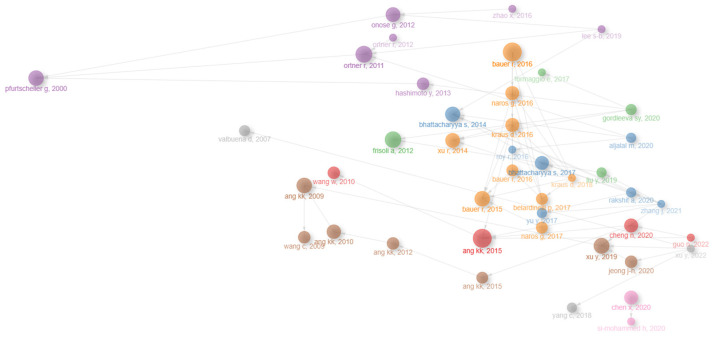
Visual representation of the historical evolution of a group of authors.

Figure 8 shows the historical evolution of authors, starting with Pfurtscheller, G. in 2000, with an increase in researchers beginning in 2009. Figure 8 shows a visual representation of a group of authors over time, with the size of the nodes indicating the number of publications or co-citations of an author, the lines indicating the co-citations, and the color indicating authors from the same field of knowledge. The clusters are connected by a common study. Some clusters have remained visible over time, indicating that the authors are working on a common topic. The pink cluster is an emerging cluster that can indicate a research trend.

**Figure 9:**
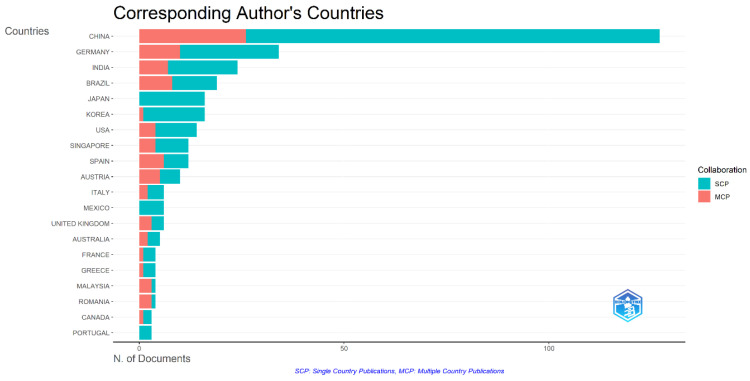
Single country publications and multi-country publications by country.

Figure 9 shows the overall cooperation among the most productive countries. Publications from the same country are identified as SCPs, while publications from several other countries are identified as MCPs. The data show that, at the time of our study, China was the most productive and cooperative country. Germany, India, and Brazil followed it in terms of productivity and international collaborations.

The corresponding author indicates the author responsible for communications with the publication source. Single country publications (SCPs) are shown in blue, with SCPs encompassing studies whose authors are affiliated with institutions from a single country. The countries of Japan, Mexico, and Portugal were found to be associated only with SCPs which did not entail the participation of authors from other countries. Multi-country publications (MCPs) are shown in orange, with the studies encompassed in this group being authored by researchers from several countries.

China, Germany, India, and Brazil were found to have the highest production levels and the highest numbers of SCP and MCP publications. Countries like South Korea, France, and Canada were found to have a high number of SCP publications and a low number of MCP publications, exhibiting a lack of greater collaboration with other countries. The United States of America, Singapore, Italy, and Australia were found to have a high number of SCP publications, although the number of MCP publications accounted for a third of the total production. Austria, Spain, and England were found to have almost the same number of SCP and MCP publications, indicating a balance between studies carried out only within the country and those carried out in collaboration with other countries. Countries like Malaysia and Romania were found to have a higher number of MCP publications compared to SCP outputs, meaning that most studies are carried out in collaboration with other countries.

**Figure 10:**
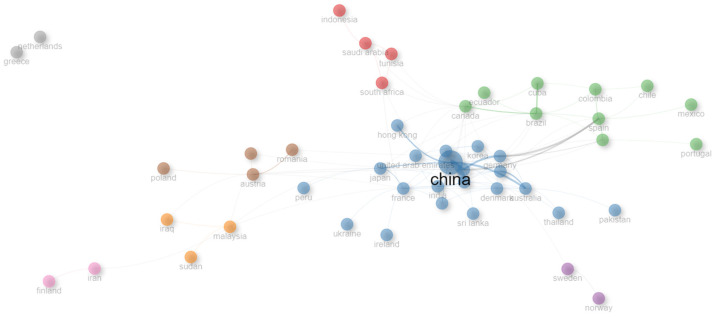
Visual representation of the collaboration network among countries.

Figure 10 shows the collaboration network that exists among different countries. It was identified that the country with the most influence in the international collaboration network is China. The network is characterized by the presence of scattered points and dense points, indicating a lack of collaboration among countries or the existence of collaboration only among certain countries. We found eight nodes identifying the groups of countries exhibiting the greatest collaboration: the first is an isolated gray node between Greece and the Netherlands, neither of which undertook any collaboration with other countries, while the other seven nodes are connected in some way. The pink node and the purple node were found to contain two countries each exhibiting the greatest degree of collaboration: Finland and Iran (pink node) and Sweden and Norway (purple node). A fourth node was found to contain three countries exhibiting a great degree of collaboration: Iraq, Malaysia, and Sudan. The brown and red nodes were found to contain four countries exhibiting great levels of collaboration, namely Romania, Austria, Turkey, and Poland (brown node) and Indonesia, Saudi Arabia, South Africa, and Tunisia (red node). A seventh node was found to contain eight collaborating countries, i.e., Canada, Ecuador, Cuba, Brazil, Colombia, Spain, Chile, Mexico, Portugal, and Italy, while the eighth node contained China, Hong Kong, United Arab Emirates, Japan, Peru, France, Ukraine, Ireland, India, Sri Lanka, Denmark, Australia, Germany, South Korea, New Zealand, Switzerland, United States of America, England, Thailand, Pakistan, and Singapore. This last node was characterized by the highest number of countries represented among the different continents. It can be observed that most of the nodes contain countries that are geographically close. The nodes containing countries exhibiting the highest degree of collaboration were found to be located in Asia–Europe–America–Oceania (node 8) and in America–Europe (node 7).

**Figure 11:**
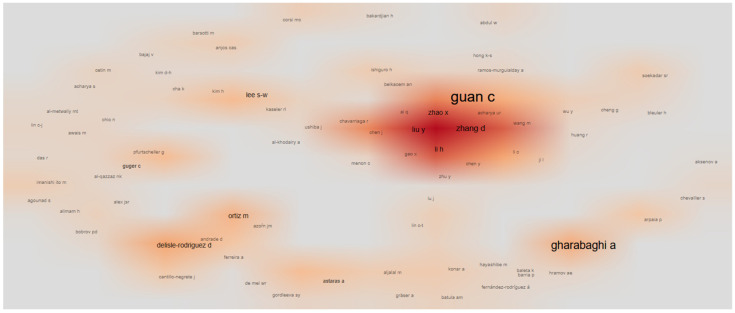
Visual representation of the heatmap of the collaboration network among authors.

Figure 11 shows that, at the time of our study, the authors exhibiting the highest degree of collaboration were Guan, C. and Gharabghi, A., followed by Zhao, X., Zhang, D., Liu, Y., Li, H., Lee, S.W., Guger, C., Delisle-Rodríguez, D., and Ortiz, M. Figure 11 shows a heatmap, which is a visual representation of the intensity of collaboration between authors. Each node is represented by an author’s name, with the font size indicating the number of collaborations in which the author has been involved. The intensity of the colors indicates a higher (dark color) or lower (light color) degree of collaboration.

Author Guan, C. has collaborated closely with the authors Zhao, X., Zhang, D., Liu, Y., and Li, H., and, in that area, the color is dark orange, meaning that the authors have collaborated on multiple publications and with a high frequency.

We also found isolated nodes, indicating less frequent collaborations, but with a large node size, such as those encompassing Delisle-Rodriguez, D., Gharabaghi, A., Ortiz, M., Lee, S.-W., and Guger, C.; these authors can be considered to be core collaborators.

#### 3.8.2. Reference Co-Citation Network

The co-citation analysis of the references, a comprehensive study based on citations and shared citations between studies, was conducted using the VOSViewer tool. This analysis generated a total of 18,506 referenced citations, each with a threshold of at least five citations from a cited reference, resulting in a graph size of 102 nodes.

**Figure 12:**
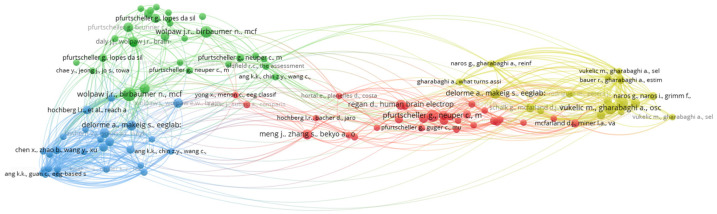
Visual representation of the co-citation reference network of studies.

Figure 12 shows the reference co-citation network. Four clusters were identified through the above analysis. Within each cluster, each circle indicates the number of citations, and the lines indicate the links between studies, in turn indicating how the reference co-citation network of studies and authors is formed. Figure 12, together with Table 8, allows for the identification of the most cited study in each cluster together with the authors associated with the highest co-citation score. In the first cluster (red), the study “Motor imagery and direct brain-computer communication”, authored by Pfurtscheller, G. and Neuper, C., was found to be associated with 16 citations and a link strength of 30 between the authors. In the second cluster (green), the study “Brain-computer interfaces for communication and control” was found to be associated with 20 citations and a link strength of 73 among the authors Wolpaw, J.R., Birbaumer, N., Mcfarland, D.J., Pfurtscheller, G., and Vaughan, T.M. In the third cluster (blue), the study “Eeglab: an open source toolbox for analysis of single-trial eeg dynamics including independent component analysis” was found to be associated with 11 citations and a link strength of 26 between the authors Delorme, A. and Makeig, S. In the fourth cluster (yellow), the study “Oscillatory entrainment of the motor cortical network during motor imagery is modulated by the feedback modality” was found to be associated with 16 citations and a link strength of 81 between the authors Vukelic, M. and Gharabaghi, A. Table 5 shows the authors Wolpaw, J.R., Birbaumer, N., Mcfarland, D.J., Pfurtscheller, G., Vaughan, T.M. (2002), Vukelic, M., Gharabaghi, A. (2015), Pfurtscheller, G., Neuper, C. (2001), Vukelic, M., Bauer, R., Naros, G., Naros, I., Braun, C., Gharabaghi, A. (2014), and Wolpaw, J.R., Birbaumer, N., Mcfarland, D.J., Pfurtscheller, G., Vaughan. T.M. (2002), who exhibited the highest number of shared citations, and authors Vukelic, M., Bauer, R., Naros, G., Naros, I., Braun, C., Gharabaghi, A. (2014), Vukelic, M., Gharabaghi, A. (2015), Wolpaw, J.R., Birbaumer, N., Mcfarland, D.J., Pfurtscheller, G., Vaughan, T.M. (2002), Bauer, R., Fels, M., Vukelic, M., Ziemann, U., Gharabaghi, A. (2015), and Schalk G., Mcfarland, D.J., Hinterberger, T., Birbaumer, N., and Wolpaw, J.R. (2004), who exhibited the highest link strength. The difference between citation frequency and link strength is that citation frequency is a count of citations, while link strength shows an author’s links compared to all possible links identified in the network [28]. Two groups of authors were identified to have contributed to the field of rehabilitation and robotics using the paradigms of MI and SSVEP. The first group of authors made contributions in 2001, 2002, and 2004, while the other group of authors made contributions in 2014 and 2015. The objective of analyzing Figure 12 and Table 8 is to identify the most representative authors with the highest reference co-citation score.

#### 3.8.3. Publication Sources Co-Citation Network

The co-citation analysis of the publication sources is based on the relationship among sources, evaluated in terms of their distance from each other, calculated using the VOSViewer tool. The relationship between the different publication sources was identified, obtaining a graph with 124 nodes.

**Figure 13:**
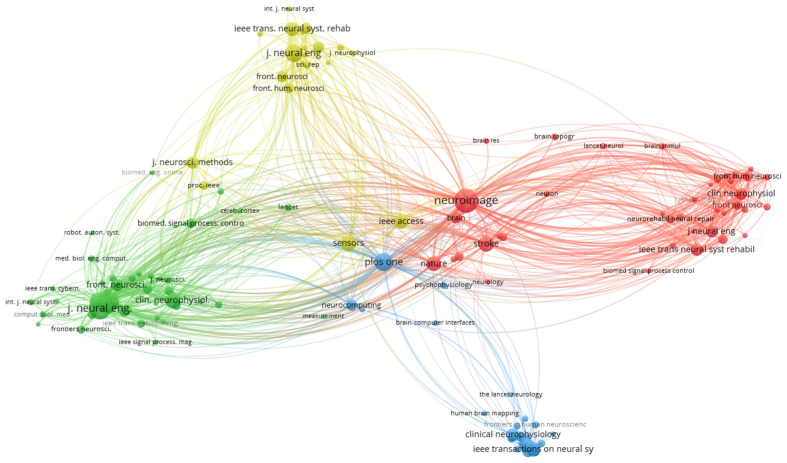
Visual representation of the co-citation network of the publication sources.

Figure 13 shows the co-citation network of the publication sources. Four clusters were identified, with each cluster led by the publication source with the highest number of co-citations. The first cluster (red) was found to encompass 39 publication sources, with the Neuroimage publication source having 471 co-citations; this journal is focused on applications related to neurorehabilitation. The cluster unites publication sources related to brain mapping using neuroimaging technologies and brain plasticity.

The second cluster (green) was found to contain 39 publication sources led by the Journal of Neural Engineering with 408 co-citations. This journal publishes studies related to the control of devices using brain–computer interfaces., and the cluster is focused on sources that publish on brain–computer interfaces and motor skill rehabilitation. The third cluster (blue) was found to contain 25 publication sources led by the Clinical Neurophysiology journal with 200 co-citations. This journal focuses on research on visually evoked potentials, and the cluster includes publication sources that publish on this paradigm and other stimulation methods. The fourth cluster (yellow) was found to contain 21 publication sources led by the IEEE Transactions on Neural Systems and Rehabilitation Engineering with 395 co-citations. This journal focuses on robotic devices and technologies for rehabilitation, and the cluster groups research related to neurorehabilitation and assisted robotics.

### 3.9. Thematic Evolution

The biblioshiny application for R-Bibliometrix was used to evaluate the thematic map by bigrams. It was reviewed in five date ranges, namely 2000 to 2009, 2010 to 2014, 2015 to 2019, 2020 to 2024, and 2000 to 2024, and four quadrants were identified: emerging, opportunity, motor, or basic. These quadrants represent distinct research areas and provide information on trends and focus areas over specific time ranges. In each map, there are nodes that are identified by specific words, with the size of the node indicating the frequency of appearance of the word in the 547 studies. If the node is large, it indicates that the associated words have had more publications related to them. Three node sizes were identified, i.e., large, medium, and small, equivalent to the frequency of appearance of the word (high, medium, and low, respectively).

**Figure 14:**
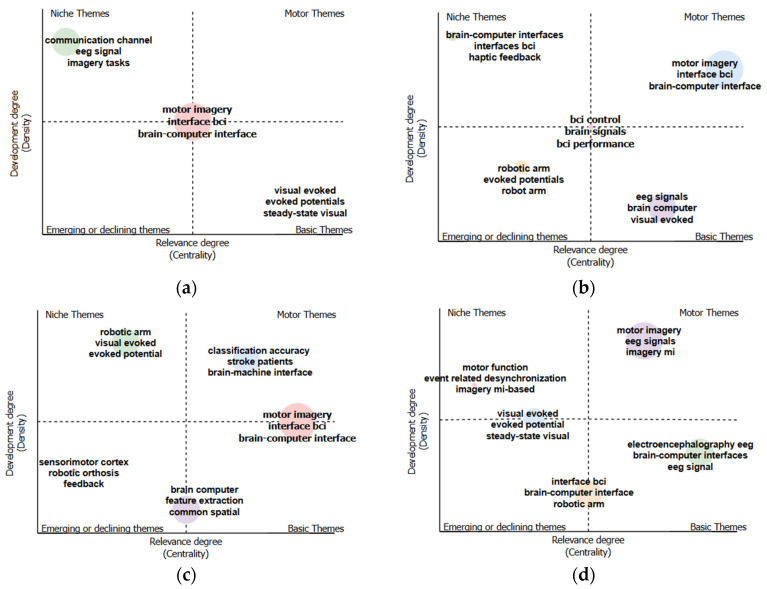
(**a**) Thematic map from 2000 to 2009. (**b**) Thematic map from 2010 to 2014. (**c**) Thematic map from 2015 to 2019. (**d**) Thematic map from 2020 to 2024.

Figure 14a shows the range from 2000 to 2009. We found that, in the opportunity quadrant, the node size was medium and that the node contained the following words: communication, EEG signal, and imagery tasks. Despite being very specific topics, the node size indicates that, in those years, there was interest in the topic, demonstrated by the number of publications containing those words. In the basic quadrant, visually evoked potentials were studied. In this quadrant, we found topics that remained stable over time and which are necessary to understand the context of the field of knowledge. The node size was found to be small, indicating a low frequency of publication and no growth. In the central point of the four quadrants, the IM and BCI paradigms were found, suggesting that, at the time of our study, the topics were experiencing a transition phase toward the motor quadrant or were experiencing a decline. Figure 14b shows the range from 2010 to 2014. We found that the motor quadrant was focused on brain–computer interface systems and on the motor imagery paradigm. The quadrant exhibited a large node size, indicating that the topics are relevant and associated with a high frequency of publication. In the opportunity quadrant, the topic of study was found to be ‘feedback’. The node was found to have a small size, indicating that the topic is new or emerging. In the basic quadrant, we found the topic to be about EEG signals, visually evoked potentials, and BCI, with a medium node size indicating that they are relevant topics that have been frequently addressed in publications. In the emerging or declining quadrant, we found the main topics to be evoked potentials, robotic arms, and BCI performance, with a small node size indicating that they are growing topics or topics in decline. In the central quadrant, we found a small node encompassing BCI control and BCI performance, indicating that the topics are in a transition phase.

Figure 14c shows the range from 2015 to 2019. We found that, in the motor quadrant, research addressed classifier performance and patients suffering from stroke and myocardial infarction. The node was found to exhibit a medium size, indicating that the topics are relevant. In the opportunity quadrant, studies were found to address visually evoked potentials and robotic arms. The node exhibited a medium size, indicating that the topics have been of great interest. In the emerging quadrant, research topics included robotic orthoses and feature extraction. The node was found to be small-sized, indicating that the topic may be growing. Two nodes were found to be in the transition zone. One node, between the emerging and basic quadrants, addressed the topics of feature extraction and common space and was found to be medium-sized, indicating that the topics may become relevant. The other node was found between the motor and basic quadrants and addressed the topics of motor imagery and BCI. The node exhibited a large size, indicating that the topics may continue to expand.

Figure 14d shows the range from 2020 to 2024. We found that, in the motor quadrant, the topic focus was on EEG signals and motor images. The node was large-sized, indicating that the topics are of great importance and have a high frequency of publication. In the basic quadrant, we found a medium-sized node addressing the topics of signaling and BCI. The medium size of the node indicates that the topics are relevant. In the opportunity quadrant, we found a small-sized node addressing the topics of event-related desynchronization and motor function. There were two medium nodes in the transition zone. The first one was found between the emerging and basic quadrants, addressing the topics of robotic arms and BCI, while, the other node was found between the niche and emerging quadrants and dealt with topics such as visually evoked potentials.

**Figure 15:**
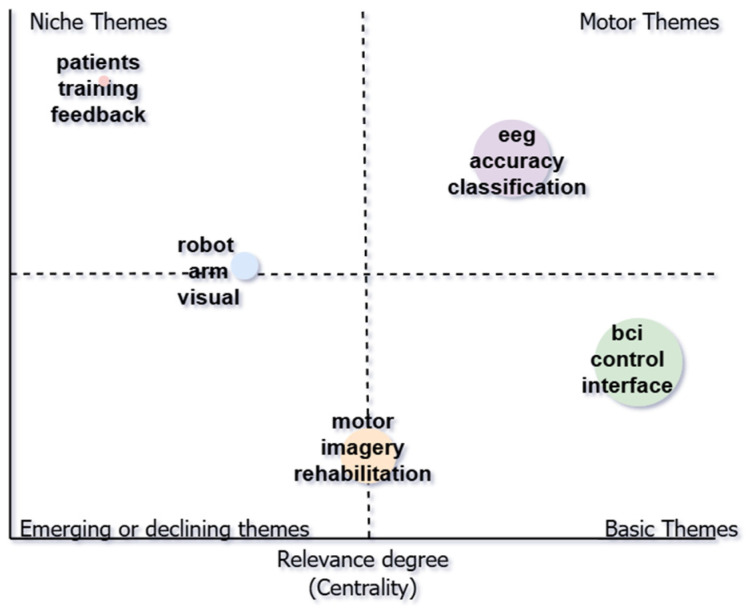
Thematic map by unigrams.

Figure 15 shows the cluster’s thematic map using unigrams from 2000 to 2024. Each of the quadrants of the Cartesian plane represents an evolutionary moment within the history of the topic. We found that, in the motor quadrant, the topic was classification, in the basic quadrant, the topics included interface and control, in the opportunity quadrant, research topics included training and feedback in patients. The node was small, indicating that the topics are emerging and are associated with a low frequency of publication. In the motor quadrant, the topics of study were eeg, accuracy, and classification. The node was medium-sized, indicating a high frequency of publication. In the basic quadrant, topics included BCI, control, and interface. The node was large, indicating relevance and a high frequency of publications. We found two nodes in the transition zone. One node was between the niche and the emerging quadrants and was small in size, while the other was between the emerging quadrant and the basic quadrant and was also small in size.

**Figure 16:**
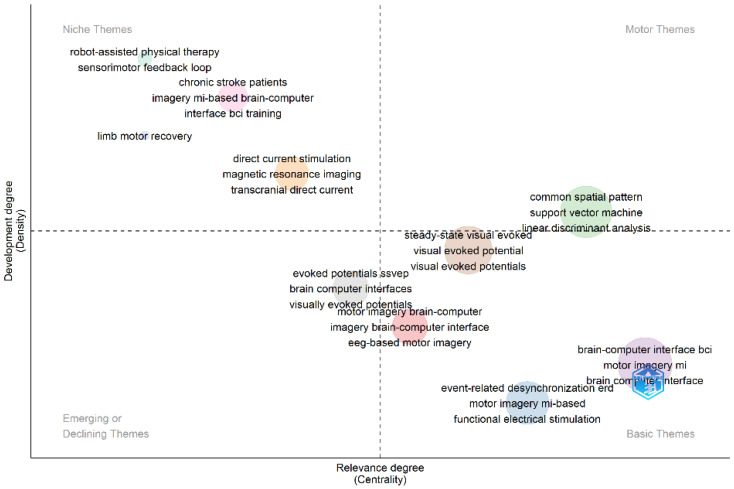
Thematic map by trigrams.

Figure 16 shows the cluster’s thematic map using trigrams. In the motor quadrant, studies addressed classification and feature extraction. In the basic and emerging quadrants, studies addressed the motor imagery paradigm and visually evoked potentials. In the opportunity quadrant, studies dealt with robotic assistance in therapies, training, motor rehabilitation of limbs, and stroke events. Nodes in the opportunity quadrant indicated areas of limited interest associated with a low publication frequency, as evidenced by the size of the small and medium nodes. Opportunity quadrant nodes encompassed areas of limited interest and associated with a low publication frequency, as evidenced by the size of the small and medium nodes. Motor quadrant nodes encompassed topics dominating the research field and which are currently critical, as evidenced by their high publication frequency. The basic quadrant contained medium-sized nodes encompassing fundamental topics in the research field associated with a high publication frequency. There were two nodes that were close to a transition zone and medium-sized, indicating that they may be moving into the basic, motor, or opportunity quadrants.

### 3.10. Co-Occurrence of Keywords

Keyword co-occurrence analysis identifies a common word across different studies in the keyword, title, or abstract fields [29].

A keyword co-occurrence analysis is performed to review the research topics using the VOSviewer tool. The Scopus database identifies the keywords, the indexing, and the sum of the previous two.

**Figure 17:**
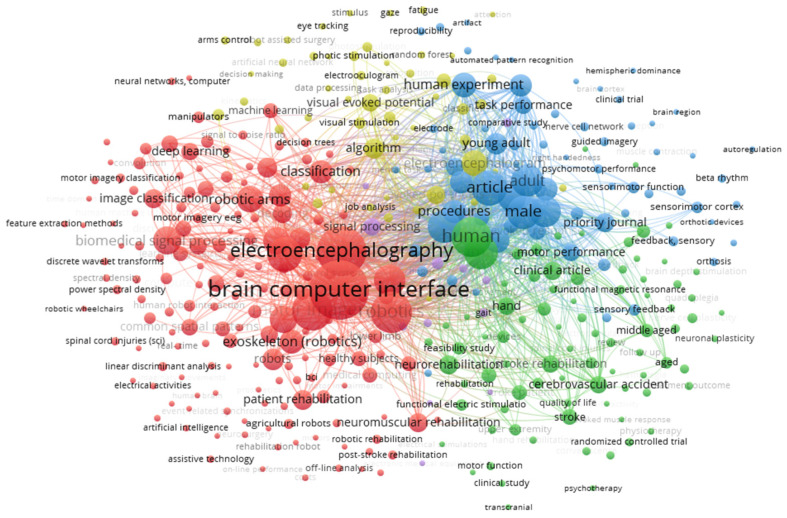
Visual representation of keyword co-occurrences.

Figure 17 shows the indexing keywords that were obtained from the 547 studies. The keywords obtained from the above studies were 3139, a co-occurrence threshold of five times per word was applied, and the graph size was limited to 381 nodes. In the network structure, five clusters were identified: cluster 1 (red) with 164 keywords, cluster 2 (green) with 83 keywords, cluster 3 (blue) with 71 keywords, cluster 4 (yellow) with 51 keywords, and cluster 5 (purple) with 12 words. The label of the most frequent keyword within the cluster suggests the thematic orientation within the cluster. In cluster 1, the focus was on brain–computer interfaces (449 occurrences), robotic applications (220 occurrences), and motor imagination (209 occurrences). In cluster 2, the focus was on human studies (356 occurrences) and the use of brain–computer interface systems in relation to neural function rehabilitation (48 occurrences) and stroke events (41 occurrences). In cluster 3, clinical studies (160 occurrences) were related to brain–computer interfaces (140 occurrences). Cluster 4 encompassed topics such as visually evoked potentials (83 occurrences) and precision in brain–computer interface systems, while cluster 5 revolved around signal processing (40 occurrences), exoskeletons (28 occurrences), and comparative studies with different technologies.

From the set of keywords, it can be concluded that work currently under development addresses the topics of robotic exoskeletons for motor rehabilitation, assisted robotics for rehabilitation or assistance in stroke patients or in relation to ALS or motor functions, as well as the development of signal processing algorithms and the use of classifiers such as support vector machines, artificial neural networks, and decision trees. We found that, with respect to classification techniques, the methods used in the analyzed studies were Fourier transform, principal component analysis, and common spatial patterns.

Each cluster has a different focus. Cluster 1 (red) identifies a growing area in brain–computer interfaces and robotic applications, cluster 2 (green) is expanding toward neurological rehabilitation in humans, cluster 3 (blue) focuses on clinical research with brain–computer interface applications, cluster 4 (yellow) focuses on technical aspects, and cluster 5 (purple) focuses on specific areas such as signal processing.

### 3.11. Data Extraction

The impact indicator selected 49 studies, and the information was organized by identifying information on the paradigm used, classification, extraction, metrics, framework, usability, application, performance, and number of participants. Of the 49 studies, 39 relied on the use of the MI paradigm, 15 studies relied on the SSVEP paradigm, and one study was hybrid.

Table 9 shows the data extracted with respect to the paradigm, application, framework, usability, and number of participants. These studies explored different paradigms, such as MI, SSVEP, and hybrid, and their applications in the fields of rehabilitation and robotics included exoskeletons, robotic arms, wheelchairs, and orthoses. While no specific framework was employed, some studies utilized frameworks for data processing. Usability was measured in the experiments, and the number of participants ranged from 1 to 49.

Table 10 shows the information related to data processing. Feature extraction used common spatial patterns (CSP), common spatial pattern filter bank (FBCSP), discrete wavelet transform (DWT), time domain parameters (TDP), power spectral density (PSD), fast Fourier transform (FFT), short-time Fourier transform, (STFT), common average referencing (CAR), independent component analysis (ICA), band power (BP), autoregressive models (AR), and canonical correlation analysis (CCA).

Classification used linear discriminant analysis (LDA), support vector machine (SVM), burst backpropagation neural networks (BPNNs), canonical correlation analysis (FBCCA), canonical correlation analysis (CCA), linear support vector machine (LSVM), kernel support vector machine (KSVM), gradient boosting (GM), regularized linear discriminant analysis (SRLDA), k-nearest neighbors (KNN), convolutional neural network at a hybrid scale (HS-CNN), random forest (RF), linear regression, logistic regression, and neural networks (NNs). The metrics used included the information transfer rate (ITR) and accuracy, the latter ranging from 45.7% to 99.11%.

Table 11 shows the information related to the software elements used to obtain brain signals, the languages used to program the algorithms, and the programs used for information processing. Hardware elements refer to the electronic, mechanical, and electrical devices used to develop the brain–computer interface system. The ‘initial assessment’ and ‘final perception’ columns refer to the experiments conducted at the beginning and end of each study, respectively, by performing additional physical, psychological, or perception tests related to the use of the brain–computer interface system.

## 4. Discussion

A bibliometric analysis is based on bibliographic data such as the evolution of publications, sources, citations, number of publications, geographical distribution, documents by topic, and production with respect to both authors and institutions.

Publications have increased over the years, with the first study being published in 2000. We found the highest number of studies to have been published in 2023, with 74 studies, followed by 2019, with 60 studies, and 2020, with 57 studies. The evolution of publications can be divided into several bands, the first comprising the years 2000, 2007, and 2008, with 1–4 studies published at an average of two studies per year. The second band extended from 2009 to 2014, with 7–19 studies published at an average of 13 studies per year. From 2015 to 2019, 27 to 60 studies were published at an average of 39 studies per year, while, from 2020 to August 2024, 41 to 74 studies were published at an average of 54 studies per year. Table 12 shows the increase in the publication rate of studies in the field of brain–computer interfaces with motor imagery and SSVEP paradigms for rehabilitation and robotic applications.

The studies published between 2000 and August 2004 tended to fall under two types of documents: 47.07% are scientific articles and 44.4% are conference proceedings or proceedings, amounting to 91.47% of all published studies, leaving 8.53% in the ‘other documents’ category.

The total number of publication sources was 309. The publication sources associated with the highest percentage of publications were the Journal of Neural Engineering, with 4.75% of all published studies, equivalent to 26 studies, and the IEEE Transactions on Neural Systems and Rehabilitation Engineering, with 4.02% of all published studies, equivalent to 22 studies. These were followed by the Proceedings of the Annual International Conference of the IEEE Engineering in Medicine and Biology Society EMBS, with 3.83% of all published studies (21 studies), and the Lecture Notes in Computer Science, including the subseries Lecture Notes in Artificial Intelligence and Lecture Notes in Bioinformatics, with 3.1% of all published studies (17 studies). There were eight publication sources (1–3%) with 7–10 publications, 60 publication sources (0.2–1%) with 2–6 publications, and 237 publication sources responsible for 0.18% studies, equivalent to a single publication. According to the metric indicators, at the time of our study, 62 publication sources were located in the Q1 quartile, with a 2003 SJR score between 0.51 and 6.09 and an H index between 11 and 503, 35 publication sources were located in the Q2 quartile, with a 2003 SJR score between 0.41 and 1.17 and an H index between 25 and 267, 24 publication sources were located in the Q3 quartile, with a 2003 SJR score between 0.13 and 0.75 and an H index between 16 and 201, 17 publication sources were located in the Q4 quartile, with a 2003 SJR score between 0.14 and 0.36 and an H index between 7 and 82, 171 publication sources with no quartile assignment but an H index between 0 and 222, and 19 publication sources with a 2003 SJR score between 0.112 and 1.62.

The bibliometric analysis identified the thematic structure of the analyzed research through the quantitative analysis of 547 studies published on brain–computer interfaces under the paradigms of motor imagery and steady-state visually evoked potentials for applications in the fields of rehabilitation and robotics. The publication source associated with the highest number of published studies was found to be the Journal of Neural Engineering from the United Kingdom, located in the Q1 quartile with an SJR-derived H index of 135 and a 2003 SJR score of 1.09. The author with the highest number of publications (20) and the highest H (16), G (20), and M (1.067) index scores was the German researcher Gharabaghi, A., from Eberhard Karls University Tübingen. Still, his production has decreased in recent years; new authors with greater production rates have emerged in recent years, such as Wang, J., Li, M., Li, Y., Wang, Y., and Jeong, J.-H. The institution with the most affiliations was found to be Eberhard Karls University Tübingen, associated with 51 studies from Germany, followed by the Institute for Infocomm Research, Singapore, and by the Korea University, South Korea. The country with the highest scientific production rate was found to be China, with 155 studies authored by 127 Chinese nationals and associated with 1994 citations, followed by the United States of America and Germany. The country with the highest number of citations was found to be Germany with 2010, followed by China and Singapore. The most cited title was “Quadcopter control in three-dimensional space using a brain-computer interface based on noninvasive motor imagery”, authored by LaFleur, K., from the United States of America, with 464 citations since 2013. Lastly, the most prolific research areas were found to be engineering and computer science (52.1%).

The co-citation network showed that the authors with the strongest relationship between ideas and authors were Schalk, G., Ang, K.K., Wolpaw, J.R., and Pfurtscheller, G., of whom Ang, K.K. started publishing in 2009 and had the third highest H (14), G (16), and M (0.875) index scoreses. Pfurtscheller, G. was identified as the first author to publish in this area in 2000. Mexico, Japan, and Portugal were found to be characterized by individual productivity. In contrast, the other countries were characterized by collaborative productivity, collaborating in particular with the following (by continent): North America (United States of America), Brazil (South America), Germany (Europe), South Africa (Africa), China (Asia), and Australia (Oceania). The collaboration network revealed the following collaborations at the continental level: Europe–Asia, America–Europe, and America–Europe–Oceania, finding that the authors involved in the most significant collaborations were Guan, C. and Gharabaghi, A., both of whom had the highest H, G, and M index scores.

The co-citation network of references identified the most referenced authors as Wolpaw, J.R., Pfurtscheller, G., and Schalk, G. The co-citation network of publication sources identified the four publication sources with the highest number of publications: Journal of Neural Engineering (United Kingdom), Clinical Neurophysiology (Ireland), IEEE Transactions on Neural Systems and Rehabilitation Engineering (United States of America), and Neuroimage (United States of America), all located in quartile Q1.

The thematic evolution began with the advent of the MI paradigm, followed by (i) the incorporation of new paradigms, such as SSVEP with the acquisition of brain signals, (ii) data processing focusing on the performance of the classifier, and, finally, (iii) applications in the context of robotic arms and brain–computer interfaces for patients with stroke. The co-occurrence of words identified the development of research in the fields of robotic exoskeletons, assisted robotics, data extraction, and classification.

Research opportunities can be found in training, patient feedback, and applications such as robot assistance in therapies, limb motor rehabilitation, and stroke.

We found a wide variety of paradigms used in rehabilitation and robotics applications, such as MI, SSVEP, and hybrid, with applications including orthoses, robotic arms, rehabilitation of upper and lower limbs, and exoskeletons. None of the documents relied on a standardized framework. During the development of the present study, it was found that some studies did work with some type of framework, particularly in the context of data processing [31,46,47,49,50,52,54,56,59,63,64,66,67,76], but some studies worked on other frameworks like BCI Graz [34], research frameworks [39], clinical frameworks [42], control frameworks [55], rehabilitation frameworks [53], and experimentation frameworks [60], but none of the studies worked with a general framework for a brain–computer interface system.

Usability was measured in the analyzed experiments, and the number of participants was variable with no standard value. However, a greater number of participants can be obtained when the study is conducted on healthy participants. If, for example, the study is conducted on participants suffering from the consequences of a stroke, the number can be decreased and personnel trained in rehabilitation or in diagnosis, with additional examinations needed to be able to carry out the experiment.

MI and SSVEP share feature extraction and classification techniques. The data processing approaches used in the analyzed experiments generally entail feature extraction techniques in the time domain such as autoregressive models [74], techniques in the frequency domain such as the Fourier transform [41,51,58,64,70] and power spectral density [35,36,50,60,70,74], and spatial techniques such as common spatial patterns [31,38,43,48,50,52,53,60,61,64,65,70,71,76]. With respect to classification techniques, we found examples of SVM [35,37,41,52,60,61,63,74], Bayesian [57] and linear discriminant analysis [34,40,47,50,52,53,60,63,65,69,74,76]. With respect to regression techniques, we found examples of linear regression and logistic regression [38,42,50,57,66,78], as well as neural networks [49,52,64,66,70,74,75]. The metrics used most commonly include accuracy and ITR with a variable performance: accuracy under the MI paradigm is between 45.7% and 96.89%, while accuracy under the SSVEP paradigm is between 60% and 99.11%.

With respect to the software elements, we found a wide variety of programs such as Matlab [32,38,42,47,50,78], BCI2000 [35,39,40,44,46], or Openvibe [69]. With respect to the initial assessment, clinical evaluations [35,38,68], initial instructions [32,69,76], initial questionnaires [33], or tests [31,42,46,51] were found in the studies, while, with respect to the final perception, few studies employed any type of final test [38,72,77].

Non-invasive paradigms allow for a variety of applications in the context of rehabilitation and robotics. Different frameworks exist for the different stages of brain–computer interface development. Depending on the type of request, participant recruitment may be a limitation when conducting research on clinical patient groups. Common feature extraction and classification techniques associated with the use of MI and SSVEP paradigms may have greater convergence, but the highly variable accuracy indicates that more research is needed to improve the reliability of processing techniques in the context of practical applications. The application of final evaluations could allow for a greater understanding of the effectiveness of the brain–computer interface from the perspective of the participants.

## 5. Conclusions

The bibliometric analysis allowed us to analyze quantitatively the 547 studies obtained from the Scopus database. Computational tools such as Scopus, biblioshiny for R-bibliometrix, and VosViewer were used for the analysis. Brain–computer interfaces were examined under the paradigms of motor imagery and steady-state visually evoked potentials for applications in the fields of rehabilitation and robotics, finding intellectual diversity based on the number of countries, authors, and sources, and a great deal of collaboration between authors and countries.

The co-citation maps showed the trends and influences of different authors, publication sources, and studies from 2000 to August 2024. The co-citation of references provided a map of the intellectual relationships between studies from the academic community’s perspective. Likewise, the citation frequency and the strength of the links were identified, finding that “Quadcopter control in three-dimensional space using a noninvasive motor imagery-based brain-computer interface” was the most referenced document at the time of our study. The authors with the highest cooperation level were found to be Guan, C. and Gharabaghi, A. The countries characterized by the highest production level (by author), cooperation, citation, and institutions were found to be China and Germany. The co-citation of authors allowed us to visualize the intellectual structure encompassing the main authors involved, finding that the strongest relationship between ideas was found among the authors Schalk, G., Ang, K.K., Wolpaw, J.R., and Pfurtscheller. G. The co-citation of publication sources provided information about the researchers and the areas of knowledge addressed by the publication sources in which the authors publish, such sources, which represent the United Kingdom and the United States of America, being located in the Q1 quartile.

The keyword co-occurrence map identified the evolution of research topics and areas of research opportunity, such as feedback, training, signal acquisition, data processing, and applications.

Data extraction identified research elements such as the paradigm used in rehabilitation and robotics applications, finding that the most used one was MI. The number of participants is not standardized, as sample sizes of 1 to 49 were found. Different feature extraction and classification techniques were identified in relation to data processing, and their evaluation was carried out with metrics such as accuracy.

This article allowed us to identify different bibliometric indicators such as the research process, evolution, visibility, volume, influence, impact, and production in relation to brain–computer interfaces under the MI and SSVEP paradigms in the fields of rehabilitation and robotics applications from the year 2000 to August 2024.

## Figures and Tables

**Table 1:** Bibliometric studies of brain–computer interfaces.

Study	Metrics	Parameters
Yin et al. (2023) [6]	Country, research area, institution, source, author, citation, and keywords	1990–2020 7880 studies
Li et al. (2023) [5]	Publication year, citation, country, institution, author, and source	2013–2022 693 studies
Said et al. (2022) [9]	List of key elements	2000–2022 18 studies
Xue et al. (2022) [8]	Number of studies, publication trends, country, institution, author, keywords, and emerging words	2010–2020 3194 studies
Torres et al. (2023) [2]	Analysis of citations, co-citations, co-authorship, and co-occurrence of keywords	2005–2021 227 studies
Yan et al. (2021) [11]	Publication year, research area, country, author, cooperation networks, and most cited studies	2006–2021 249 studies
Stegman et al. (2020) [3]	Co-citations	2000–2018 756 studies
Nobrega et al. (2019) [10]	Type of document, research area, and country	2009–2019 226 studies
Hu et al. (2016) [7]	Citations, trends, and patterns	2006–2015 100 studies
Hamadicharef (2010) [4]	Publication year, citation, and source	1998–2008 1081 studies

**Table 2:** Metadata analysis conducted by using R-bibliometrix.

Metadata	Description	Status
AB	Abstract	Excellent
DT	Document type	Excellent
SO	Journal	Excellent
LA	Language	Excellent
PY	Publication year	Excellent
TI	Title	Excellent
TC	Total citation	Excellent
AU	Author	Good
C1	Affiliation	Good
CR	Cited references	Good
DI	DOI	Good
ID	Keyword plus	Good
DE	Keywords	Acceptable

**Table 3:** Number of studies per publication source and publication source metrics.

Publication Source	Studies	SJR-H-INDEX	SJR-2023	SJR-Quartile
Journal of Neural Engineering	26	135	1.09	Q1
IEEE Transactions on Neural Systems and Rehabilitation Engineering	22	164	1.32	Q1
Proceedings of the Annual International Conference of the IEEE Engineering in Medicine and Biology Society EMBS	21	40	0.34	
Lecture Notes in Computer Science Including Subseries Lecture Notes in Artificial Intelligence and Lecture Notes in Bioinformatics	17		0.606	
Communications in Computer and Information Science	10	69	0.2	Q4
Frontiers in Human Neuroscience	9	157	0.79	Q2
IEEE International Conference on Rehabilitation Robotics	9	45	0.24	Q3
Biomedical Signal Processing and Control	8	108	1.28	Q1
ACM International Conference Proceeding Series	7	151	0.25	
Frontiers in Neuroscience	7	153	1.06	Q2
IFMBE Proceedings	7	37	0.14	
Lecture Notes in Electrical Engineering	7	45	0.15	Q4
Frontiers in Neurorobotics	5	50	0.68	Q2
IEEE Transactions on Cognitive and Developmental Systems	5	55	1.3	Q1
Neuroimage	5	418	2.44	Q1
PLoS ONE	5	435	0.84	Q1
Sensors Switzerland	5	245	0.79	Q1

**Table 4:** Author classification based on the H, G, and M indexes.

Author	H_Index	G_Index	M_Index	NP	PY_Start
Gharabaghi, A.	16	20	1.067	20	2010
Guan, C.	15	17	0.938	17	2009
Ang, K.K.	14	16	0.875	16	2009
Phua, K.S.	12	14	0.75	14	2009
Wang, C.	12	16	0.75	16	2009
Lee, S.-W.	9	12	0.75	12	2013
Liu, Y.	9	12	0.9	12	2015
Zhang, D.	8	12	0.615	12	2012
Guger, C.	7	10	0.28	10	2000
Li, M.	7	11	0.583	11	2013
Li, Y.	7	11	0.7	11	2015
Jeong, J.-H.	6	10	0.857	10	2018
Wang, Y.	6	11	0.857	11	2018
Konar, A.	5	10	0.455	10	2014
Wang, J.	5	12	0.556	12	2016

**Table 5:** Number of studies per institution.

Institution	Studies	Country
Eberhard Karls University Tübingen	51	Germany
Institute for Infocomm Research	41	Singapore
Korea University	41	South Korea
Federal University of Espirito Santo (UFES)	38	Brazil
Shangai Jiao Tong University	30	China
Aristotle University of Thessaloniki	26	Greece
Miguel Hernández University of Elche	26	Spain
South China University of Technology	25	China
Tianjin University	25	China
Tianjin University of Technology	25	China

**Table 6:** Scientific production, authors, and number of citations by country.

Country	Scientific Production	Authors	Number of Citations
China	155	127	1994
USA	47	14	487
Germany	46	34	2010
India	40	24	322
Japan	34	16	376
Spain	32	12	120
South Korea	30	16	427
Brazil	25	19	453
Singapore	23	12	961
Austria	17	10	860
Italy	21	6	365

**Table 7:** Number of citations per study.

Study	Year	Cite
Quadcopter control in three-dimensional space using a noninvasive motor imagery-based computer interface	2013	464
Brain oscillations control hand orthosis in a tetraplegic	2000	391
A Randomized Controlled Trial of EEG-Based Motor Imagery Brain-Computer Interface Robotic Rehabilitation for Stroke	2015	384
Long-Term Training with a Brain-Machine Interface-Based Gait Protocol Induces Partial Neurological Recovery in Paraplegic Patients	2016	313
Brain-computer interface-based robotic end effector system for wrist and hand rehabilitation: Results of a three-armed randomized controlled trial for chronic stroke	2014	254
An SSVEP BCI to control a hand orthosis for persons with tetraplegia	2011	247
HS-CNN: A CNN with hybrid convolution scale for EEG motor imagery classification	2020	222
Resting state changes in functional connectivity correlate with movement recovery for BCI and robot-Assisted upper-extremity training after stroke	2013	215
Clinical study of neurorehabilitation in stroke using EEG-based motor imagery brain-computer interface with robotic feedback	2010	198
Brain-Controlled Robotic Arm System Based on Multi-Directional CNN-BiLSTM Network Using EEG Signals	2020	192
A new gaze-BCI-driven control of an upper limb exoskeleton for rehabilitation in real-world tasks	2012	181
A lower limb exoskeleton control system based on steady state visual evoked potentials	2015	181
Proprioceptive feedback and Brain Computer Interface (BCI) Based Neuroprostheses	2012	181
Brain-machine interfaces for controlling lower-limb powered robotic systems	2018	170
Neural Interface Technology for Rehabilitation: Exploiting and Promoting Neuroplasticity	2010	167
Closing the sensorimotor loop: Haptic feedback facilitates decoding of motor imagery	2011	164
Review on motor imagery based BCI systems for upper limb post-stroke neurorehabilitation: From designing to application	2020	151
Deep learning techniques for classification of electroencephalogram (EEG) motor imagery (MI) signals: a review	2023	150

**Table 8:** Ranking of most co-cited references based on citations and link strength.

Author	Cite	Total Link Strength
Wolpaw. J.R., Birbaumer, N., Mcfarland, D.J., Pfurtscheller, G., and Vaughan, T.M. (2002)	20	73
Vukelic, M. and Gharabaghi, A. (2015)	16	81
Pfurtscheller, G. and Neuper, C. (2001)	16	30
Vukelic, M., Bauer, R., Naros, G., Naros, I., Braun, C., and Gharabaghi, A. (2014)	15	89
Wolpaw, J.R., Birbaumer, N., Mcfarland, D.J., Pfurtscheller, G., and Vaughan, T.M. (2002)	15	21
Bauer, R., Fels, M., Vukelic, M., Ziemann, U., and Gharabaghi, A. (2015)	9	67
Schalk, G., Mcfarland, D.J., Hinterberger, T., Birbaumer, N., Wolpaw. J.R. (2004)	9	63

**Table 9:** General data extracted from the investigated studies.

Study	Paradigm	Application	Framework	Usability	Participants
Pfurtscheller et al. (2000) [30]	MI	Orthosis	-	Daily training, 2–4 sessions (60 min)	1
Ang et al. (2009) [31]	MI	Robotic rehabilitation	Extraction—classification	Pre-assessment	18
Lee J. et al. (2009) [32]	MI	Robotic arm	-	Visual information	3
Mandel et al. (2009) [33]	SSVEP	Wheelchair	-	Pre-use wheelchair study	9
Müller-Putz et al. (2010) [34]	MI	Robotic arm	BCI Graz	Online experiment	10
Gomez-Rodriguez et al. (2011) [35]	MI	Neurorehabilitation	-	Experiment (training—online)	8
Ortner R. et al. (2011) [36]	SSVEP	Orthosis	-	Online experiment	7
Frisoli et al. (2012) [37]	MI	Upper limb neurorehabilitation	Robotic control	Online experiment	7
Onose et al. (2012) [38]	MI	Robotic arm	-	Patient’s perception of control	9
Ramos-Murguialday et al. (2012) [39]	MI	Exoskeleton	Research framework	Online experiment	24
LaFleur et al. (2013) [40]	MI	Quadcopter	-	Drone control training	5
Sakurada et al. (2013) [41]	SSVEP	Upper limb rehabilitation	-	Online experiment	15
Várkuti et al. (2013) [42]	MI	Rehabilitation	Clinic framework	-	9
Ang et al. (2014) [43]	MI	Rehabilitation	-	Online experiment	21
Gharabaghi et al. (2014) [44]	MI	Neurorehabilitation	-	Online experiment	1
Van Dokkum et al. (2014) [45]	MI	Rehablitiation	-	Experiment (training)	20
Witkowski et al. (2014) [46]	MI	Hand exoskeleton	Classification	Online experiment	12
Xu R. et al. (2014) [47]	MI	Orthosis	Classification	Online experiment	9
Ang et al. (2015) [48]	MI	Robotic feedback	-	Experiment (training—online)	19
Kwak et al. (2015) [49]	SSVEP	Lower limb exoskeleton	Classification	Online experiment	11
Yong et al. (2015) [50]	MI	Robotic arm	Extraction—classification	Online experiment	12
Li et al. (2016) [51]	SSVEP	-	-	Online experiment	28
Tang et al. (2016) [52]	MI	Upper limb exoskeleton	Extraction—classification	Experiment (training—control online)	4
Zhao et al. (2016) [53]	SSVEP	Upper limb rehabilitation	Rehabilitation framework	Online experiment	5
Gao et al. (2017) [54]	Hybrid (MI-SSVEP)	Robotic arm	Extraction	Writing experiment	8
Gui et al. (2017) [55]	SSVEP	Exoskeleton	Control framework	-	6
Chen X. et al. (2018) [56]	SSVEP	Robotic arm	Extraction—sistema SSVEP–BCI	Online experiment	12
He et al. (2018) [57]	MI	Lower limb exoskeleton	Prisma	-	54
Yang et al. (2018) [58]	SSVEP	Robotic manipulator	Stimuli—classification	Baxter robot experiment	2
Chen X. et al. (2019) [59]	SSVEP	Robotic arm	Extraction—classification	Online experiment	10
Lee et al. (2019) [60]	MI	Robotic arm	Experiment, data analysis, and application	-	13
Taran et al. (2019) [61]	MI	Robotic arm	Framework for task classification	Online experiment	-
Apaia et al. (2020) [62]	SSVEP	Rehabilitation	-	Online experiment	20
Chu et al. (2020) [63]	MI	Robotic arm	Classification	Online experiment	12
Dai et al. (2020) [64]	MI	Classification	Classification	BCI competition IV	18
Gordleeva et al. (2020) [65]	MI	Upper limb exoskeleton	-	Experiment (training—online)	8
Jeong et al. (2020) [66]	MI	Robotic arm	Classification	Online experiment	15
Ke et al. (2020) [67]	SSVEP	Robotic Arm	Classification	CS–BCI and AR–BCI experiment	14
Khan et al. (2020) [68]	MI	FES, robotic, and virtual reality	-	Clinical evaluations and comparison between experimental and control groups	49
Si-Mohammed et al. (2020) [69]	SSVEP	Movil robot	-	Feedback	37
Altaheri et al. (2021) [70]	MI	Assistance and rehabilitation	Prisma	-	-
Baniqued et al. (2021) [71]	MI	Hand rehabilitation	Prisma	-	208 in 11 studies
Chen et al. (2021) [72]	SSVEP	Robotic arm	ROS operating system framework	Online experiment	10
Li et al. (2021) [73]	SSVEP	Robotic arm	-	Online experiment	1
Zhang et al. (2021) [74]	MI	Assistance and rehabilitation	Exoskeleton framework	Online experiment	-
Ak et al. (2022) [75]	MI	Robotic arm	-	Experiment (training—online)	4
Cho et al. (2022) [76]	MI	Robotic hand	Classification	Online experiment	12
Xu et al. (2022) [77]	MI	Robotic arm	Strategic control framework	Online experiment	-
Brunner et al. (2024) [78]	MI	Upper limb rehabilitation	-	Experiment (training—online)	40

**Table 10:** Data processing.

Study	Extraction	Classification	Metrics	Performance
Pfurtscheller et al. (2000) [30]	-	-	Accuracy	65–95%
Ang et al. (2009) [31]	FBCSP	-	Accuracy	55.6%
Lee J. et al. (2009) [32]	-	-	-	-
Mandel et al. (2009) [33]	-	-	Accuracy	88.68%
Müller-Putz et al. (2010) [34]	Laplacian	LDA	Accuracy	83.8%
Gomez-Rodriguez et al. (2011) [35]	PSD	SVM	AUC	0.63–0.76
Ortner R. et al. (2011) [36]	PSD	-	Accuracy	60%
Frisoli et al. (2012) [37]	-	SVM	-	-
Onose et al. (2012) [38]	CSP	Linear regressions	Accuracy	70.5–81%
Ramos-Murguialday et al. (2012) [39]	-	-	-	-
LaFleur et al. (2013) [40]	-	LDA	ITR	-
Sakurada et al. (2013) [41]	FFT, CCA	SVM	Accuracy	80–88%
Várkuti et al. (2013) [42]	ICA	Linear regression	-	-
Ang et al. (2014) [43]	FBCSP	-	-	-
Gharabaghi et al. (2014) [44]	-	-	-	-
Van Dokkum et al. (2014) [45]	-	-	-	-
Witkowski et al. (2014) [46]	-	-	-	-
Xu R. et al. (2014) [47]	-	LDA	TPR (true positive rate), FP (false positives)	60–70%
Ang et al. (2015) [48]	FBCSP	-	Accuracy	57–62.9%
Kwak et al. (2015) [49]	CCA	CCA, KNN	Accuracy	91.3%
Yong et al. (2015) [50]	CSP, FBCSP, BP	LDA, logistic regression, SVM	Accuracy	60.7–66.9%
Li et al. (2016) [51]	FFT	CCA	-	-
Tang et al. (2016) [52]	CSP	LDA, SVM, BPNN	Accuracy	87.37–84.29%
Zhao et al. (2016) [53]	CSP	LDA	Accuracy	73.9%
Gao et al. (2017) [54]	DWT	CCA	ITR	0.73–0.93
Gui et al. (2017) [55]	-	-	Accuracy	92.4%
Chen et al. (2018) [56]	CCA	FBCCA	Accuracy, ITR	92.78%
He et al. (2018) [57]	CAR, CCA	Logistic regression, LDA, Bayesian classifier	ITR	0.37 bit/s
Yang et al. (2018) [58]	FFT	CCA	-	-
Chen X. et al. (2019) [59]	CCA	CCA, FBCCA	Accuracy, ITR	97.75%
Lee et al. (2019) [60]	CSP, TDP, PSD	LVSM, KSVM, GM, SRLDA	Accuracy	45.7–91.4%
Taran et al. (2019) [61]	CSP	SVM	Accuracy	96.89%
Apaia et al. (2020) [62]	CCA	CCA	Accuracy	92.6%
Chu et al. (2020) [63]	Riemannian geometric framework	LDA, SVM	Accuracy	80.5–79.7%
Dai et al. (2020) [64]	STFT, CSP, FBCSP	HS-CNN	Accuracy	80%
Gordleeva et al. (2020) [65]	CSP	LDA	Accuracy	78.3–83.4%
Jeong et al. (2020) [66]	-	CNN, SVM, multiple linear regression	-	-
Ke et al. (2020) [67]	CCA	CCA, FBCCA	Accuracy	CS-BCI: 98.57–99.11%, AR-BCI: 87.68–97.59%
Khan et al. (2020) [68]	-	-	Accuracy	95%
Si-Mohammed et al. (2020) [69]	-	LDA	Accuracy	75–80%
Altaheri et al. (2021) [70]	CSP, FBCSP, FFT, PSD	Deep learning	Accuracy	68.4–77.34%
Baniqued et al. (2021) [71]	CSP	-	-	-
Chen et al. (2021) [72]	CCA	CCA	Accuracy	78.59–95.27%
Li et al. (2021) [73]	-	-	Accuracy	87.5%
Zhang et al. (2021) [74]	ICA, PSD, AR	LDA, SVM, KNN, RF, NN	Accuracy	94.5%
Ak et al. (2022) [75]	Time–frequency and non-linear techniques	CNN	Accuracy	85–92.59%
Cho et al. (2022) [76]	CSP	LDA	-	-
Xu et al. (2022) [77]	-	-	-	-
Brunner et al. (2024) [78]	-	Logistic regression	-	-

**Table 11:** Software and hardware elements and initial and final evaluations.

Study	Software	Hardware	Initial Assessment	Final Perception
Pfurtscheller et al. (2000) [30]	-	Orthosis, EEG 60 channels	-	-
Ang et al. (2009) [31]	-	Nuamps EEG, unipolar electrodes AgAgCl	Fugl–Meyer scores.	-
Lee J. et al. (2009) [32]	Matlab	Robotic arm, clinical scanner	Initial instructions.	-
Mandel et al. (2009) [33]	Navigation software	Wheelchair	Initial questionnaire.	-
Müller-Putz et al. (2010) [34]	Matlab	Ag/AgCl 32 channels	-	-
Gomez-Rodriguez et al. (2011) [35]	BCI2000	Robotic arm 7 DOF, EEG 35 channels	Clinical evaluation.	-
Ortner R. et al. (2011) [36]	-	Orthosis, Guger Technologies	-	-
Frisoli et al. (2012) [37]	-	Kinect sensor, GusBamp 13 channels	Training phase.	-
Onose et al. (2012) [38]	Matlab	Robotic arm, cameras, 64 channel amplifier	Clinical functional examinations and medical assistance.	Personalized follow-up questionnaire and patients’ perception of the controllability of the EEG-BCI.
Ramos-Murguialday et al. (2012) [39]	BCI2000	EasyCap 128 channels	Electroencephalogram before the experiment.	-
LaFleur et al. (2013) [40]	BCI2000	Drone	-	-
Sakurada et al. (2013) [41]	-	9 DOF, DC servomotors, adjustable link lengths for upper arm, forearm, and fingers	-	-
Várkuti et al. (2013) [42]	Matlab	Nuamp 27 channels	Fugl–Meyer scores.	-
Ang et al. (2014) [43]	-	Nuamp, haptic robot	Ability to operate the BCI and Fugl–Meyer scores.	-
Gharabaghi et al. (2014) [44]	BrainVision, BCI2000	Orthosis, EEG 32 channels	-	-
Van Dokkum et al. (2014) [45]	-	-	-	-
Witkowski et al. (2014) [46]	BCI2000	ActiCap, BrainAmp	Edinburgh manual dominance quiz.	-
Xu R. et al. (2014) [47]	Matlab	Magstim 200, Servo controlled hydraulic actuator	-	-
Ang et al. (2015) [48]	Nuamps	EEG neuroscan, nuamps, robot	Ability to operate the MI-BCI and Fugl–Meyer motor assessment scores.	-
Kwak et al. (2015) [49]	-	Atmega 128, GmbH	-	-
Yong et al. (2015) [50]	Matlab	Geodesic net amps	-	-
Li et al. (2016) [51]	-	Gtec, robotic arm	They closed their eyes before the experiment.	-
Tang et al. (2016) [52]	EEG system Active Two 64 channels	One degree of freedom exoskeleton	-	-
Zhao et al. (2016) [53]	-	Epoc EEG	-	-
Gao et al. (2017) [54]	-	Emotiv EPOC, robotic arm	-	-
Gui et al. (2017) [55]	-	Emotiv EPOC, exoskeleton	-	-
Chen et al. (2018) [56]	Wincaps III	Robotic arm 7 DOF	-	-
He et al. (2018) [57]	-	Exoskeleton	-	-
Yang et al. (2018) [58]	DirectX	Camera, neuroscan 40 channels, robotic manipulator	-	-
Chen X. et al. (2019) [59]	-	Camera, robotic arm	-	-
Lee et al. (2019) [60]	-	Robotic arm and hand	-	-
Taran et al. (2019) [61]	-	-	-	-
Apaia et al. (2020) [62]	C, Android app	Raspberry pi 3, smart glasses	-	-
Chu et al. (2020) [63]	-	Ag/AgCl 64 channels	-	-
Dai et al. (2020) [64]	-	-	-	-
Gordleeva et al. (2020) [65]	-	Amplifier NVX52	Participants were interviewed to determine the dominant leg before the experiments.	-
Jeong et al. (2020) [66]	-	-	Participants had to meet the following criteria: normal health, enough sleep, no alcohol, no heavy exercise.	-
Ke et al. (2020) [67]	3D Unity	Microsoft HoloLens	-	-
Khan et al. (2020) [68]	-	Gtec 16 channels, Recoverix, robotic arm	Clinical evaluation.	-
Si-Mohammed et al. (2020) [69]	Openvibe	HoloLens, robot	Initial instructions.	-
Altaheri et al. (2021) [70]	-	-	-	-
Baniqued et al. (2021) [71]	-	2–256 electrodes, Ag/AgCl	-	-
Chen et al. (2021) [72]	-	-	-	Comfort questionnaire.
Li et al. (2021) [73]	-	Ag/AgCl	-	-
Zhang et al. (2021) [74]	-	-	-	-
Ak et al. (2022) [75]	-	-	-	-
Cho et al. (2022) [76]	-	-	Initial instructions.	-
Xu et al. (2022) [77]	-	BrainAmp 32 channels	-	Work score.
Brunner et al. (2024) [78]	Matlab	Recoverix, FES devices, 16 active electrodes	-	-

**Table 12:** Number of studies by year.

Year	Average Studies	Increase Studies
2000–2008	2	-
2009–2014	12.5	525%
2015–2019	39.4	215.2%
2020–2024	53.8	36.54%
